# Extensive Presentation of Central Ossifying Fibroma Treated with Conservative Surgical Excision

**DOI:** 10.1155/2014/204258

**Published:** 2014-11-23

**Authors:** Matheus Henrique Lopes Dominguete, Alexandre Augusto Sarto Dominguette, Bruno Henrique Matos, Paulo Roberto Dominguete, Jorge Esquiche León, Lucinei Roberto Oliveira

**Affiliations:** ^1^Universidade Vale do Rio Verde (UninCor), Avenida Castelo Branco 82, Chácara das Rosas, 37410-000, Três Corações, MG, Brazil; ^2^Faculdade de Odontologia de Ribeirão Preto (FORP/USP), Ribeirão Preto, SP, Brazil

## Abstract

Central ossifying fibroma is a benign slow-growing tumor of mesenchymal origin and it tends to occur in the second and third decades of life, with predilection for women and for the mandibular premolar and molar areas. Clinically, it is a large asymptomatic tumor of aggressive appearance, with possible tooth displacement. Occasionally treated by curettage enucleation, this conservative surgical excision is showing a recurrence rate extremely low. The objective of this study was to report a case of a 44-year-old woman, presenting a very large ossifying fibroma in the mandible, which was successfully treated with curettage, and to conduct a brief literature review of this lesion, focusing on the histology, clinical behavior, and management of these uncommon lesions.

## 1. Introduction

Central ossifying fibroma (COF) is the most common benign fibroosseous neoplasm of the oral and maxillofacial region. It was described by Menzel in 1872 but was appointed by Montgomery in 1927 [1]. This lesion tends to occur in the second and third decades of life, commonly in women, and in the mandibular premolar and molar areas [1–8].

Central ossifying fibroma usually presents clinically as a painless and expansive spherical or ovoid jawbone mass that may displace the roots of adjacent teeth and cause root resorption [9, 10]. COF demonstrates either completely radiolucent or a mixture of radiolucent and radiopaque appearance (depending on the amount of internal calcification) [10, 11], and it is histologically composed of proliferating fibroblasts and osseous products that include bone and cementum-like material. However, there are some other lesions of the maxillary bones that should be included in the differential diagnosis, such as focal cementum-osseous dysplasia, osteoid osteoma, and fibrous dysplasia [12, 13]. Most COFs have a good prognosis and can be treated by conservative surgical excision through the use of curettage, enucleation, or excision [1, 2, 11, 12].

The aims of this report are to present a case of COF in the mandible and to provide a critical review of the current literature regarding lesions of this type.

## 2. Case Report

**Figure 1:**
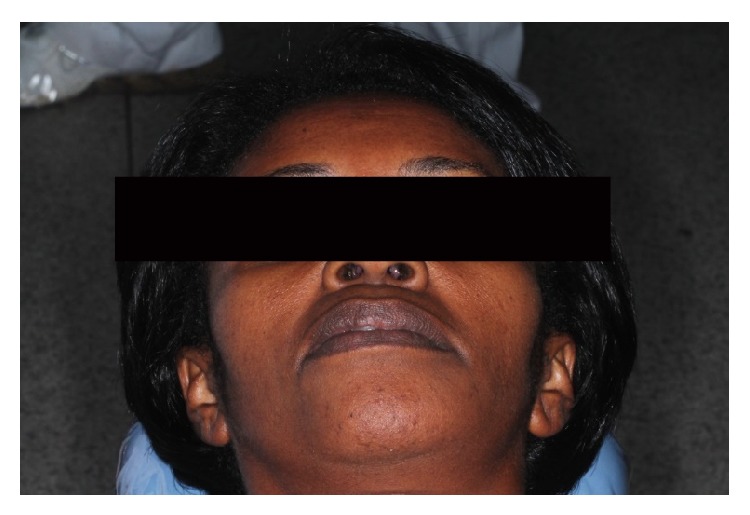
Preoperative clinical appearance of the patient.

**Figure 2:**
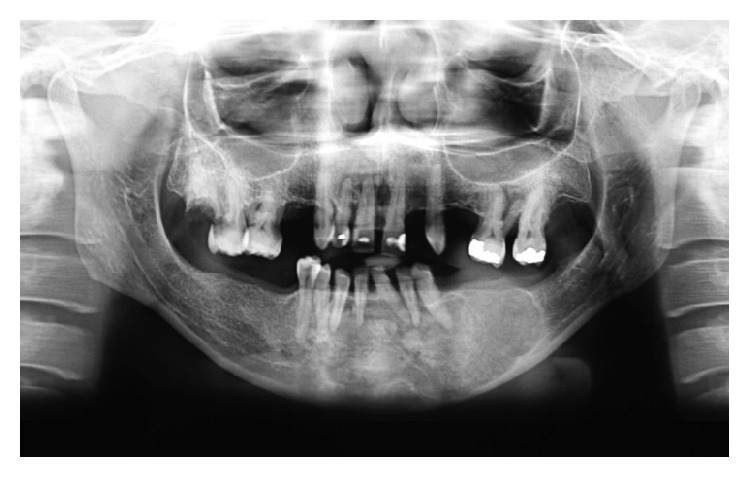
Panoramic radiograph indicates the left mandibular multilocular radiolucent lesion.

A 44-year-old black woman was referred to the maxillofacial surgery clinic of the Vale do Rio Verde University for evaluation of an asymptomatic swelling in the vestibular region of the left mandible canine (Figure 1). The exact date when the swelling was observed was not known. The overlying mucosa was normal in color, and the needle aspiration yielded negative results. In addition, no lymphadenopathy was found on the extraoral examination. Analysis of the panoramic radiograph revealed a lesion with a mixed radiopaque and radiolucent appearance, a well-circumscribed border from the right lateral incisor to the left second molar region, and a diameter of approximately 10 cm (Figure 2). There was radicular resorption of tooth 41. An incisional biopsy of the lesion was performed, and, based on the clinical findings, imaging results, and histopathologic features, a diagnosis of COF was confirmed. Under local anesthesia, a curettage enucleation of the tumor was performed, combined with the extraction of odontogenic remnants. The removed tumor was then sent again for histopathologic study, and the results confirmed the original diagnosis (Figures 7 and 8). After a twelve-month follow-up, no recurrence of this lesion was observed in this patient (Figure 9).

## 3. Discussion

COF is more frequent in female patients in the second to fourth decades of life [3, 10, 16–18]. Reports on where these tumors are most frequently localized differ, with some identifying the maxilla as the most frequent site [5, 13]. However, the most commonly reported site is the mandible, especially in the molar region [9], as was observed in the present case.

Most cases of COFs are asymptomatic, with the first clinical manifestation being a swelling of the mandibular cortical layer, which produces a marked extraoral facial asymmetry [9, 10]. The case presented herein showed clinical features similar to those of previously reported cases.

When the lesion occurs in patients between 5 years and 15 years of age, it is called a juvenile ossifying fibroma [19], which differs from the reported case, because the patient is in the fourth decade of life, and shows the central variant of the central ossifying fibroma.

The differential diagnosis of COF is based on radiographic features. As a completely radiolucent lesion, COF can be misdiagnosed as a lesion with a radiographically similar appearance, such as focal cementoosseous dysplasia, odontogenic cyst, periapical granuloma, traumatic bone cyst, unilocular ameloblastoma, and central giant cell granuloma. The imaging features in the present case were similar to those of most reported cases, showing a circumscribed radiolucent lesion with well-defined margins and intralesional calcification [7, 9].

Histologically, COFs contain ossicles that connect to form bone trabeculae that are usually surrounded by osteoblasts and occasionally by osteoclasts. Cementum-like rounded calcifications are also frequently observed, and a mixture of these two types of calcifications is commonly observed inside a single lesion (Figure 5) [1, 2, 20, 21].

Enucleation by curettage has been reported as a method for the treatment of COF (Table 1). In some reports, the authors favor conservative surgery rather than en bloc resection. In these cases, conservative curettage is completed until healthy bony margins are reached [20, 21]. Some cases treated by conservative surgical excision have shown no recurrence over a 17-year follow-up period [15]. In contrast, Zama et al. (2004) reported an immediate recurrence 15 days after conservative surgery to treat COF of the mandible, which required a second operation for hemimandibulectomy and reconstruction [21].

The clinical management of COF remains uncertain. To avoid or minimize the chance of recurrence, a partial or en bloc resection of the jaw is preferred for larger lesions [17, 22]. Although our reported case had a large lesion, the surgical protocol applied was conservative because the lesion was well circumscribed and could be separated from normal bone during surgery, and the current follow-up has not shown any clinical signs of recurrence.

**Figure 3:**
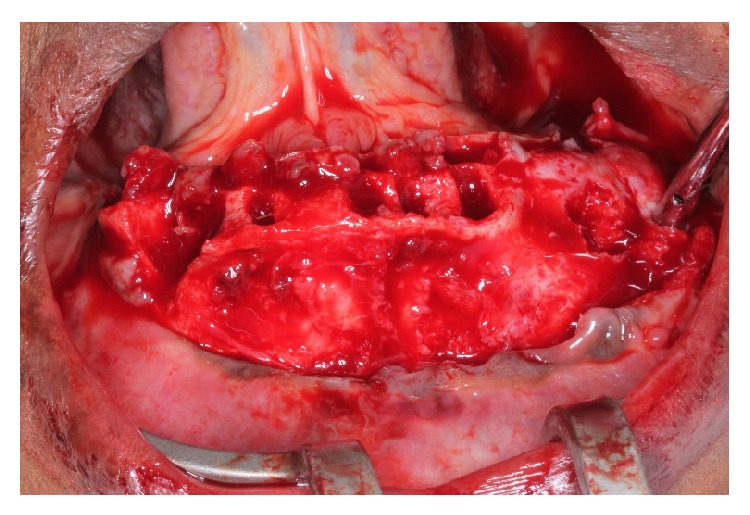
Surgical access to the injured area and the removal of all lower teeth.

**Figure 4:**
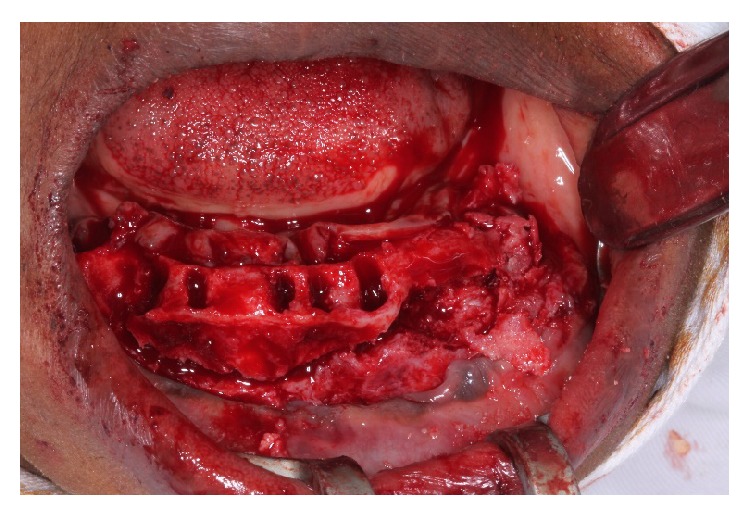
Removal of the lesion by enucleation with curettage.

**Figure 5:**
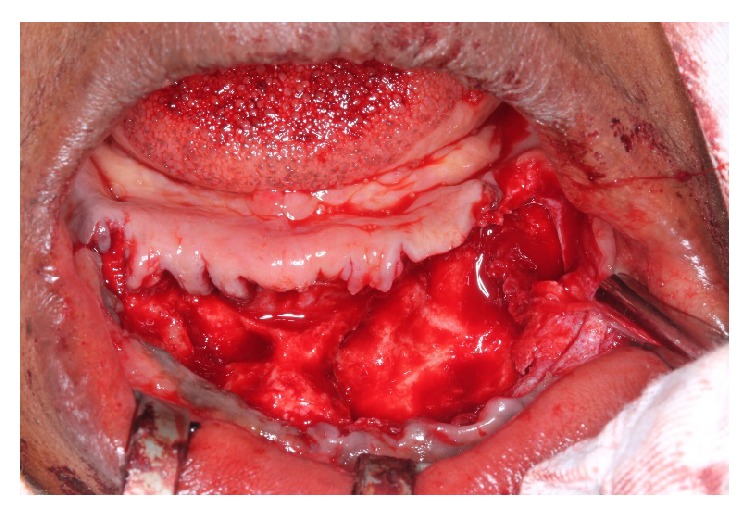
Surgical site after removal of the entire lesion.

**Figure 6:**
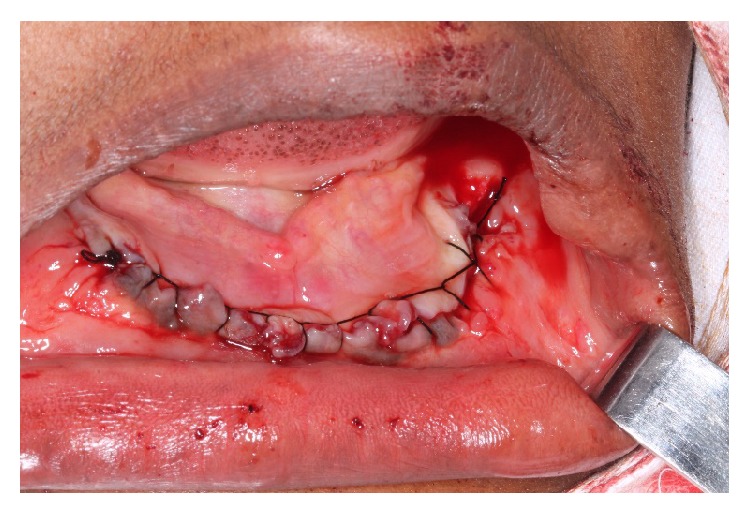
Suturing of the surgical site.

**Figure 7:**
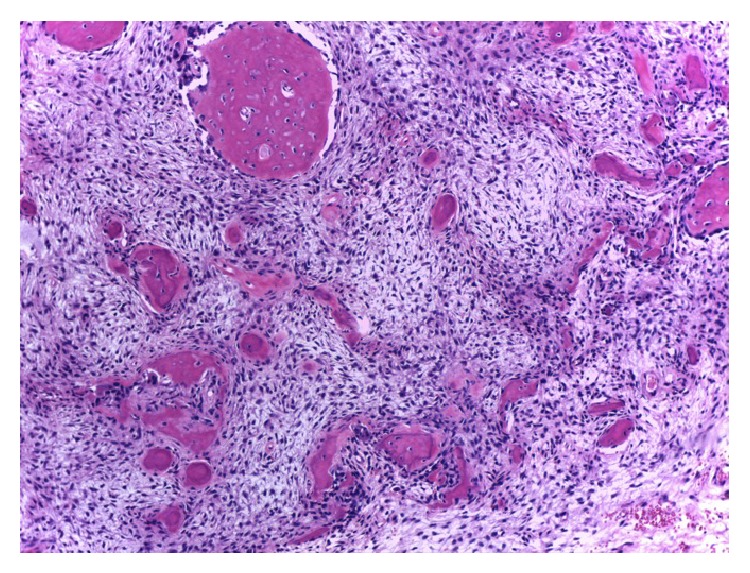
Histopathologic appearance of the ossifying fibroma showing fibroblastic stroma with small calcifications (hematoxylin-eosin, 10x).

**Figure 8:**
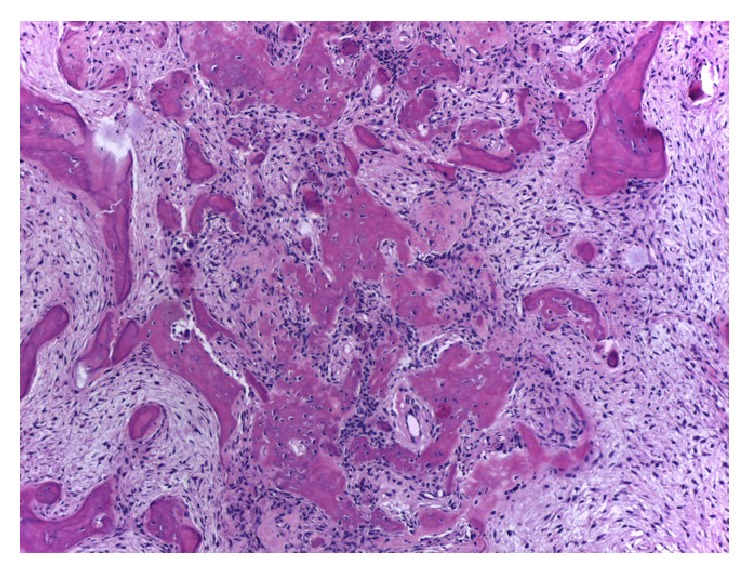
Histopathologic appearance of a central ossifying fibroma shows spherules of cementoid material in a highly cellular fibrous connective tissue stroma (hematoxylin-eosin, 10x).

**Figure 9:**
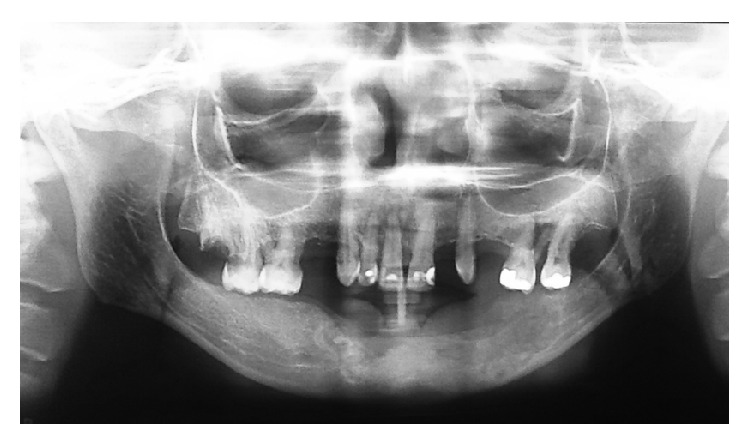
Panoramic radiograph 12 months after the surgery.

Our findings showed that the conservative surgical excision of COF appears to be a versatile, secure, and satisfactory treatment option (Figures 3 and 4). Although the recurrence rate of this tumor appears to be extremely low, patients must be followed carefully, particularly because the tumor has proved to be aggressive and to occasionally recur after conservative surgical procedures (Figure 6).

## Figures and Tables

**Table 1:** Characteristics of the cases treated by curettage reported in the literature.

Case	Author	Genre/age	Location	Treatment	Follow-up
1	Liu et al. [2]	F/45	Left mandible	Curettage	
2	Liu et al. [2]	M/15	Right mandible	Curettage	
3	Bertolini et al. [14]	F/37	Left mandible	Curettage	
4	Triantafillidou et al. [15]	M/28	Right mandible	Curettage	Recurrence after 2 years
5	Triantafillidou et al. [15]	F/7	Right maxilla	Curettage	Recurrence after 6 months
6	Triantafillidou et al. [15]	F/16	Right mandible	Curettage	17 years free of recurrence
7	Triantafillidou et al. [15]	F/14	Left mandible	Curettage	17 years free of recurrence
8	Triantafillidou et al. [15]	M/54	Right mandible	Curettage	11 years free of recurrence
9	Triantafillidou et al. [15]	F/41	Right mandible	Curettage	9 years free of recurrence
10	Triantafillidou et al. [15]	F/43	Right mandible	Curettage	4 years free of recurrence
11	Triantafillidou et al. [15]	F/37	Right mandible	Curettage	3 years free of recurrence
12	Triantafillidou et al. [15]	M/55	Right mandible	Curettage	2 years free of recurrence
